# Actions of Camptothecin Derivatives on Larvae and Adults of the Arboviral Vector *Aedes aegypti*

**DOI:** 10.3390/molecules26206226

**Published:** 2021-10-15

**Authors:** Frederick A. Partridge, Beth C. Poulton, Milly A. I. Lake, Rebecca A. Lees, Harry-Jack Mann, Gareth J. Lycett, David B. Sattelle

**Affiliations:** 1 Centre for Respiratory Biology, UCL Respiratory, Division of Medicine, University College London, London WC1E 6BT, UK; f.partridge@ucl.ac.uk (F.A.P.); milly.lake123@hotmail.co.uk (M.A.I.L.); hjmann3@gmail.com (H.-J.M.); 2 Liverpool School of Tropical Medicine, Pembroke Place, Liverpool L3 5QA, UK; Beth.Poulton@lstmed.ac.uk (B.C.P.); rebeccaannelees@gmail.com (R.A.L.)

**Keywords:** insecticide, mosquito, *Aedes*, camptothecin, vector, rubitecan

## Abstract

Mosquito-borne viruses including dengue, Zika, and Chikungunya viruses, and parasites such as malaria and *Onchocerca volvulus* endanger health and economic security around the globe, and emerging mosquito-borne pathogens have pandemic potential. However, the rapid spread of insecticide resistance threatens our ability to control mosquito vectors. Larvae of *Aedes aegypti* were screened with the Medicines for Malaria Venture Pandemic Response Box, an open-source compound library, using INVAPP, an invertebrate automated phenotyping platform suited to high-throughput chemical screening of larval motility. We identified rubitecan (a synthetic derivative of camptothecin) as a hit compound that reduced *A. aegypti* larval motility. Both rubitecan and camptothecin displayed concentration dependent reduction in larval motility with estimated EC_50_ of 25.5 ± 5.0 µM and 22.3 ± 5.4 µM, respectively. We extended our investigation to adult mosquitoes and found that camptothecin increased lethality when delivered in a blood meal to *A. aegypti* adults at 100 µM and 10 µM, and completely blocked egg laying when fed at 100 µM. Camptothecin and its derivatives are inhibitors of topoisomerase I, have known activity against several agricultural pests, and are also approved for the treatment of several cancers. Crucially, they can inhibit Zika virus replication in human cells, so there is potential for dual targeting of both the vector and an important arbovirus that it carries.

## 1. Introduction

### 1.1. Vector-Borne Diseases and Pandemics

Humans have had to contend repeatedly with disease epidemics throughout history. Viruses such as Ebola, HIV, SARS-CoV-2, and Zika underscore the vulnerability of the human population to emerging pathogens. Furthermore, changes in our environment and society such as urbanization, increased travel, and climate change make epidemics more frequent and harder to control [1]. New and emerging infectious diseases, together with problems of antimicrobial resistance, are a challenge to our limited anti-infective medications and other tools for controlling diseases. To help to address this problem, the Medicines for Malaria Venture recently launched the Pandemic Response Box, an open-source drug discovery program, where laboratories around the world collaborate by screening a library of structurally diverse compounds selected for potential activity against infective and neglected diseases.

Diseases transmitted by arthropod vectors endanger people in many areas of the globe. These vector-borne pathogens include protozoa, such as *Plasmodium*, *Trypanosoma*, and Leishmania; nematodes, such as Onchocerca volvulus; as well as viruses, such as Chikungunya, dengue, yellow fever and Zika [2]. These diseases infect hundreds of millions of people, malaria kills 600,000 people each year, and Dengue kills 40,000 [3,4]. The 2015 Zika epidemic, where the virus, spread via the mosquito *Aedes aegypti*, was introduced into the Americas and then spread rapidly, infecting perhaps 500,000 people, underscores vividly the pandemic potential of vector-borne diseases [5].

### 1.2. Control of Disease Vectors

An important method for reducing the impact of vector-borne diseases is to target the vector. In the case of malaria, the incidence of clinical disease fell by 40% between 2000 and 2015, and it was estimated that over half of this reduction was due to insecticide-treated nets (ITNs) that target the disease-transmitting Anopheles adult mosquitoes [6]. However, ITNs have limitations, in particular the growing resistance to pyrethroids and other insecticides [7]. ITNs are less useful for the control of pathogens spread by *A. aegypti*, which include chikungunya, dengue, yellow fever, and Zika, as the mosquitoes prefer to feed outdoors at dawn and dusk. Larval source management is also important for vector control. This targets the larval stages of mosquitoes, which in the case of *A. aegypti* develop primarily in artificial, aquatic, urban environments, such as used tires, drains, and sewers, with the aim of reducing the prevalence of the adult vector. Application of mosquito larvicides is an important component of larval source management. The major classes of larvicides in current use are chemical insecticides, typically organophosphates, such as temephos, the sodium channel targeting pyrethroids, bacterial larvicides such as *Bacillus thuringiensis* toxin, which impact gut cell membrane permeability, and insect growth regulators, like diflubenzuron, which block development of the insect.

However, resistance to current larvicides is a problem, with, for example, *A. aegypti* resistance to temephos, the major organophosphate larvicide, widespread in Brazil [8]. Whilst pyrethroid use in water sources is now prohibited because of toxicity to fish [9], pyrethroids used in agriculture are known to leach into aquatic ecosystems. Even at the low concentrations observed, early larval exposure is thought to exacerbate the development of pyrethroid-resistance in adults [10] in areas where mosquito control is needed, [11] and also that such larval stressors can impact the adult immune response [12]. Identification and development of new larvicidal agents thus remains a priority.

### 1.3. Open Science and the MMV Pandemic Response Box

Open science is an alternative way of doing science that aims to open up the research process, making innovation more efficient by the timely sharing of data, creation of collaborative communities, and avoiding duplication of effort [13,14]. An example is the Medicines for Malaria Venture (MMV) Pandemic Response Box project, an open-source distributed drug-discovery project, where a compound library is screened in multiple laboratories in a diversity of assays. The goal is identification of small molecules with potential for development to control emerging diseases with pandemic potential. It follows on from the successful MMV Malaria and Pathogen Box projects [15,16].

We developed a screening platform, INVAPP, that quantifies movement or growth of an organism in microplates [17]. This system was originally developed to search for new anthelmintics [18,19,20,21]. We recently adapted this platform for screening mosquito larvae of various species [22]. Here, we report the use of the INVAPP platform as part of the Medicines for Malaria Venture Pandemic Response Box project by screening for new anti-mosquito compounds that could be useful in the control of vector-borne diseases.

## 2. Results

**Figure 1:**
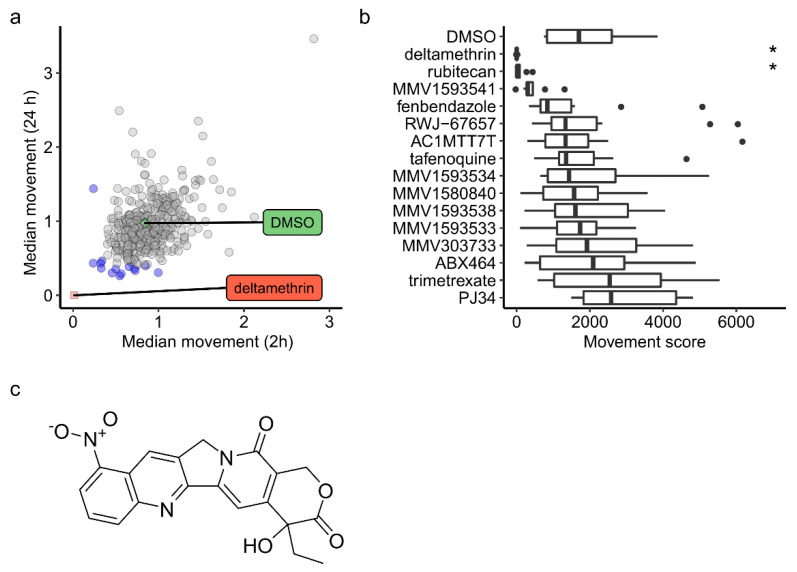
Screening 400-compound MMV Pandemic Response Box chemical library in an *A. aegypti* larval motility assay led to identification of hit compound rubitecan. (**a**) Primary screen. Each point is effect of one compound on motility at 2 h and 24 h, normalized to motility at 0 h timepoint. *n* = 3. DMSO-only and deltamethrin were negative and positive control compounds, respectively. Blue points indicate 14 compounds that were selected as candidate hit compounds. (**b**) Secondary screen, showing effects of each compound on motility after 24 h. *n* = 10. * indicates *p* < 0.05 compared to that of DMSO-only control (Dunnett’s test). Dots indicate individual outlying datapoints beyond the boxplot whiskers. (**c**) Structure of rubitecan.

The actions on *A. aegypti* larval motility of each of the 400 compounds in the Pandemic Response Box was measured using the INVAPP system at 0, 2, and 24 h timepoints. Figure 1a shows the effects of each compound tested, as well as DMSO-only and deltamethrin controls, on motility at 2 h and 24 h. Deltamethrin at 10 µM effectively paralyses the larvae, but some compounds in the library showed some reduction in larval motility. The data for all 400 compounds in the MMV Pandemic Response Box are provided in Table S1. Fourteen compounds, highlighted in blue in Figure 1a, that reduced motility at 2 h and/or 24 h to less than 40% of DMSO-only controls were selected as candidate hits and taken forward to a secondary screen. These compounds were retested at 10 µM in a secondary screen (Figure 1b), where the effects on motility after 24 h of treatment were analysed. A one-way ANOVA test found a significant effect of compound treatment on motility [F(15,144) = 3.891 *p* = 7.86 × 10^−6^. Dunnett’s test was then used to compare each compound with the DMSO-only control. Deltamethrin (the positive control, *p* = 0.027) and rubitecan (*p* = 0.041) showed a significant difference in motility compared to that of the control. The structure of rubitecan is shown in Figure 1c. Rubitecan is a topoisomerase inhibitor, originally developed as a potential therapy for various cancers [23].

**Figure 2:**
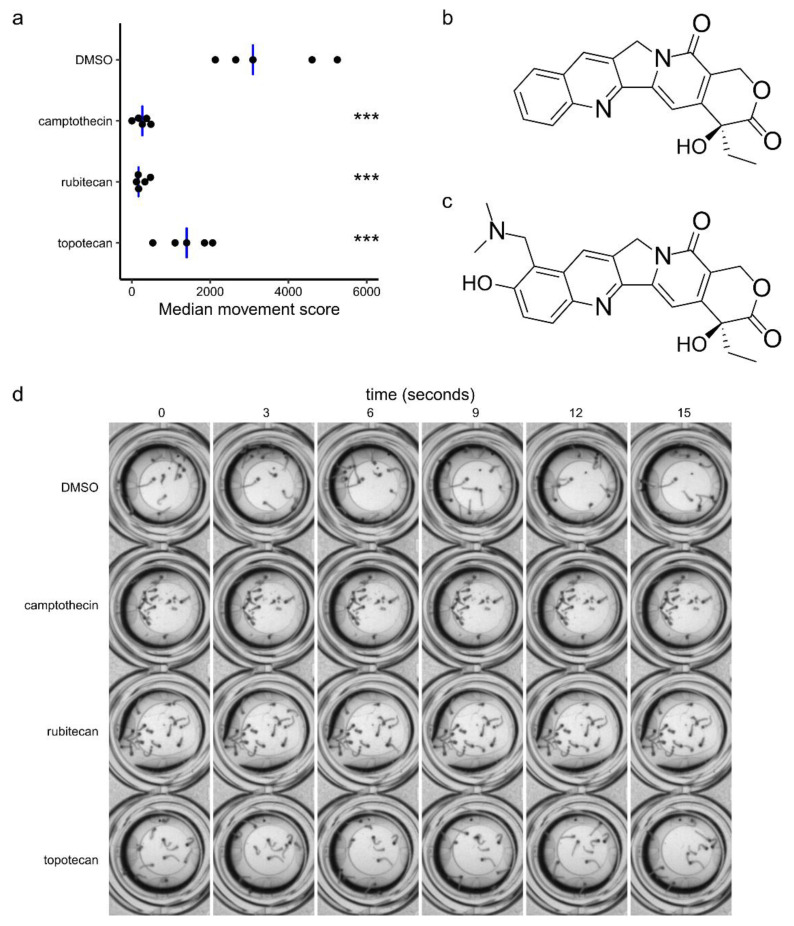
Retesting actions on larval motility of rubitecan and related compounds prepared freshly from solid material**.** (**a**) Retesting in *A. aegypti* larval motility assay of rubitecan prepared from solid material and testing of two related compounds, camptothecin, and topotecan, also prepared from solid material. Compounds were screened at 100 µM. Black dots indicate the within-batch median movement score for each of *n* = 5 biological replicates (batches of independently hatched mosquito larvae). Blue bars indicate between-batch median. A one-way ANOVA showed a significant effect of compound treatment F(3,16) = 22.0 *p* = 6.32 × 10^−6^. A posthoc Dunnett’s test was then used to compare compound treatments with the DMSO-only control. *** indicates *p* < 0.001. (**b**) Structure of camptothecin. (**c**) Structure of topotecan. (**d**) Time-lapse montage of representative assay wells. This is presented as video in Video S1.

Having pursued these initial studies using library material stored as DMSO stocks, it was important to confirm the activity of rubitecan in the larval motility assay using solutions freshly prepared from solid material. Rubitecan is a synthetic derivative of camptothecin (Figure 2b), an alkaloid isolated from *Camptotheca acuminata*, a tree native to China. We also wanted to determine the activity of camptothecin itself, as well as topotecan (Figure 2c), another camptothecin derivative, which is approved for the treatment of cervical, ovarian, and small cell lung cancers. These compounds were tested in the same 24 h treatment larval motility assay at 100 µM. These results are shown in Figure 2a. A one-way ANOVA showed a significant effect of treatment, F(3,16) = 22.0 *p* = 6.32 × 10^−6^. Dunnett’s test was then used to compare each treatment with the DMSO-only control. Camptothecin (*p* = 8.5 × 10^−6^), rubitecan (*p* = 8.3 × 10^−6^), and topotecan (*p* = 0.00087) all showed a significant difference in motility compared to that of the control, although the effect on motility was less in the case of topotecan. Examples of mosquito morphology and movement in wells treated with each compound are presented in Video S1. A time-lapse montage is also shown in Figure 2d.

**Figure 3:**
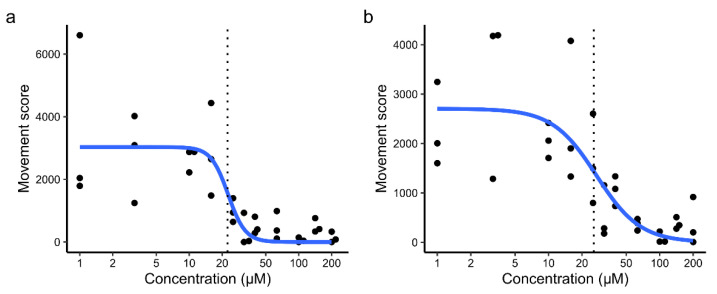
Concentration dependence of actions on larval motility of (**a**) camptothecin and (**b**) rubitecan. Curve fitted using the 4-factor log-logistic model. *n* = 3. Each point shows the measured movement score for a single well of mosquito larvae treated with the indicated concentration. Dotted lines indicate EC_50_.

We next wanted to determine the concentration dependence of the larvicidal effect of the hit compounds. Concentration-response curves were obtained using the same larval motility assay (Figure 3a,b). Curves were fitted using the 4-factor log-logistic model. The EC_50_ of camptothecin was estimated to be 22.3 ± 5.4 µM, and that of rubitecan was estimated to be 25.5 ± 5.0 µM.

**Figure 4:**
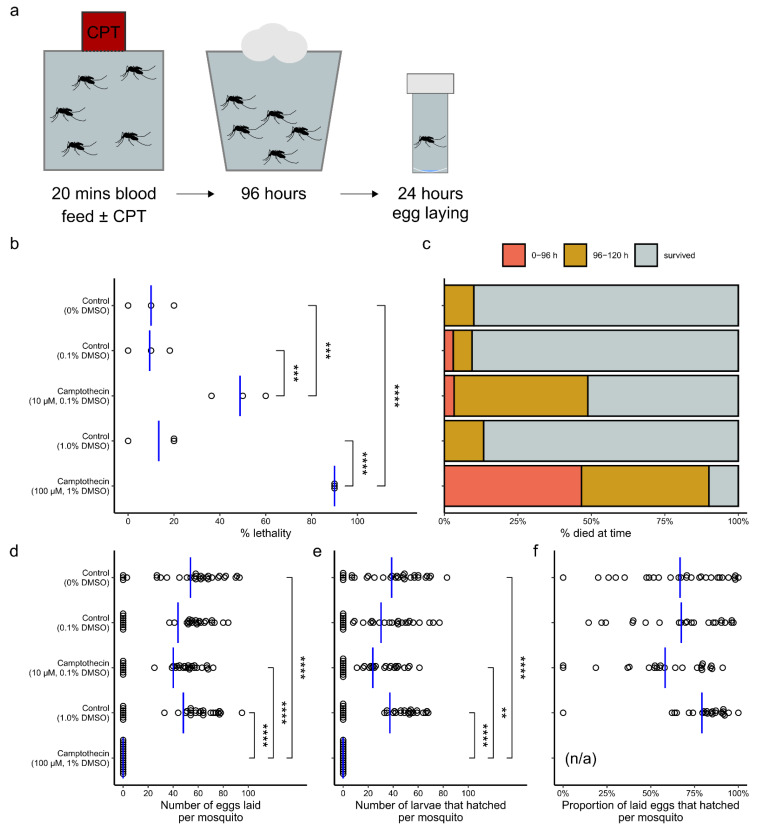
Assessing effects of camptothecin on adult *A. aegypti.* (**a**) Diagrammatic representation of methodological process. (**b**) Effect of camptothecin concentration on percentage lethality across entire experiment—each point represents percentage lethality in each experimental replicate (*n* = 3). (**c**) Effect of camptothecin concentration on percentage of total females which died indicating period during experiment when death occurred (red = 0–96 h, yellow = 96–120 h) or survived to end of the experiment (grey). (**d**) Effect of camptothecin concentration on number of eggs laid per individual mosquito. Each point represents number of eggs laid by an individual female (*n* = 30 per condition). (**e**) Effect of camptothecin concentration on number of larvae hatched per individual mosquito. Each point represents number of larvae hatched from an individual female (*n* = 30 per condition). (**f**) Effect of camptothecin concentration on hatch percentage. Each point represents the proportion of eggs which hatched for each individual female (*n* = 30 per condition). No females laid eggs and so no hatch percentage could be calculated where (n/a) is noted. Vertical blue lines indicate mean and significance as determined using a Tukey posthoc assessment is indicated as follows on (**b**,**d**–**f**) (**** < 0.0001, *** < 0.001, ** < 0.01, ’absence of bracket’ > 0.05).

Finally, we investigated the usefulness of camptothecin to target adult mosquitoes. Attempts to kill adult mosquitoes in tarsal assays (100 μM, 30 min) and in sugar meals (100 μM, daily) did not indicate a strong phenotype (data not shown). Females were fed with blood containing camptothecin according to the regimen outlined in Figure 4a, and mortality was recorded at each time point. The results are shown in Figure 4b,c. There was a significant difference in lethality between treatment groups as determined by one-way ANOVA (F(4,10) = 40.72, *p* = 3.7 × 10^−6^). A Tukey posthoc test (95% CI ± 25.7) revealed significant increased mortality in females fed 100μM camptothecin compared to that of those fed no compound (+80.0%, *p* = 9.8 × 10^−6^), 1% DMSO (+76.7%, *p* = 1.45 × 10^−5^) and 10 μM camptothecin (+41.2%, *p* = 0.0026) and in those fed 10 μM camptothecin compared to that of those fed no compound (+38.8%, *p* = 0.004) and 0.01% DMSO (+39.4%, *p* = 0.0036).

We also measured the number of eggs laid per adult mosquito (Figure 4d), the number of larvae that hatched per adult mosquito (Figure 4e), and the proportion of eggs that hatched for each treated adult mosquito (Figure 4f). Significant differences in the number of eggs laid were also observed between treatment groups using a one-way ANOVA (F(4,131) = 12.52, *p* = 1.16 × 10^−8^). Females exposed to 100 μM camptothecin did not lay any eggs. A Tukey post hoc (95% CI ± 22.19) indicated significant differences in the number of eggs laid by females fed 100 μM camptothecin and females fed no compound (−53.9 eggs, *p* < 1 × 10^−7^), 1% DMSO (−48.13 eggs, *p* = 2 × 10^−7^), and 10 μM camptothecin (−40.1 eggs, *p* = 1.81 × 10^−5^).

The number of larvae that hatched also differed significantly by treatment using a one-way ANOVA (F(4,131) = 9.846, *p* = 5.38 × 10^−7^), but the significant reductions observed with a posthoc Tukey (95%CI ± 18.9) were between females fed 100 μM camptothecin and females fed no compound (−38.7 larvae, *p* = 9 × 10^−7^), 1% DMSO (−37.4 larvae, *p* = 2.3 × 10^−6^), and 10 μM camptothecin (−23.7 larvae, *p* = 0.00632).

No significant differences in egg laying and number of larvae hatched were observed between females fed 10 μM camptothecin and controls, and no significant differences in larval hatch percentage between treatments were detected using a one-way ANOVA (F(3,87) = 2.687, *p =* 0.0514).

## 3. Discussion

### 3.1. Camptothecin Derivatives as Insecticides

In this study, we screened the MMV Pandemic Response box in a mosquito larval motility assay, and identified that camptothecin, as well as the derivatives rubitecan and topotecan, had antilarval activity against *A. aegypti.* This observation is concordant with previous observations of camptothecin-related compounds having insecticidal properties, although no compound from this chemotype reached the market for this use [24]. Indeed, a crude extract of *C. acuminata* was traditionally used in China to control pests [25]. Camptothecin was first shown to have chemo-sterilant activity against the housefly [26], and camptothecin or derivative compounds are active against agricultural pests [27,28].

This study used larvae 24 h after commencement of hatching, as these small larvae are suited for high-throughput larvicide screening due to their compatibility with 96-well plates and dispensing by pipette or microplate filler. Larvicidal activity may differ between different instars [29]. Therefore, further investigation of camptothecin derivatives should utilize additional larval stages.

### 3.2. Camptothecin Derivatives as Antivirals

Camptothecin derivatives, including topotecan and irinotecan, were approved for the treatment of various cancers. They are inhibitors of topoisomerase I (TOP1), an enzyme important for DNA replication and repair, as well as transcription. Because of this mechanism, camptothecin derivatives were investigated as potential antivirals [30]. Of particular note, camptothecin or derivatives have shown activity in cells against herpes simplex virus type 2 [31] and enterovirus 71 [32]. Interestingly, camptothecin also suppresses the host response to viral and bacterial infection and protected mice in a model of lethal inflammation [33].

The dual insecticidal and antiviral activities of camptothecin-like compounds is intriguing and may motivate further study. Zika virus is transmitted vertically within the *A. aegypti* population [34,35], and vertical transmission in the mosquito host also occurs with many other flaviviruses. Furthermore, *A. aegypti* larvae can acquire Zika virus from the environment, such as sewage containing the virus, and are able to transmit the virus to mammalian hosts [36]. Camptothecin, 1-hydroxycamptothecin, irinotecan, and topotecan were shown to inhibit Zika virus replication in human cells [37]. Therefore, a camptothecin-based antilarval compound may also have a role to play in reducing viral transmission by acting on the virus in the insect.

### 3.3. Safety for Larvicidal Use

**Figure 5:**
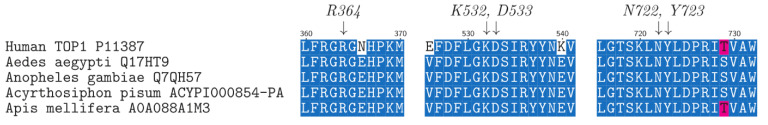
Conservation of camptothecin binding site in topoisomerase I between insects and vertebrates. Residues of TOP1 that make direct contact with topotecan in a crystal structure [38] are highlighted above alignment.

Camptothecin and related compounds are cytotoxic to mammalian cells, which underpins their use in chemotherapy. We do not underestimate the challenge of deploying such compounds in the environment as larvicides. The target of camptothecin, topoisomerase I, is a highly conserved enzyme, with all the residues that contact the drug in a topotecan/human topoisomerase I crystal structure [38] conserved across insects (Figure 5). This likely limits our ability to make more insect-specific derivative molecules by exploiting differences in target binding.

However, camptothecin itself did not find use in humans due to problems with pharmacokinetics. These problems include limited water solubility, rapid ring opening in plasma, where the active lactone is converted to an inactive carboxylate in plasma, and variable bioavailability, preventing oral dosing of camptothecin [39,40]. The route to clinical approval involved the development of water-soluble analogues of camptothecin, of which the first to be approved was topotecan, for intravenous administration, in 1996. Topotecan has appreciable oral bioavailability, around 35–40% [41], and 10-fold greater stability as the active lactone form in human blood compared to that of camptothecin [42]. Topotecan was approved for oral administration in 2007. Interestingly, in our mosquito assay, topotecan was much less active than camptothecin. This suggests that there is potential for identification of camptothecin derivatives that have acceptable safety profiles by exploiting pharmacokinetic differences between target insects and people, such as drug access or metabolism. Organophosphates, currently important mosquito larvicides, have a poor safety record. Organophosphate poisoning, either due to occupational exposure or self-harm, kills an estimated 200,000 people each year [43]. This underscores the need to develop safer effective mosquito larvicides.

### 3.4. Use as an Adulticide

Females fed 100 µM and 10 µM camptothecin in a blood meal demonstrated significantly increased mortality (90% and 48.8% respectively) across the experiment compared to that of controls. Absolute blocking of egg laying was observed in females fed 100 µM camptothecin but no effect on egg laying, larval hatching, or hatch percentage was observed for those fed 10 µM and many laid eggs prior to death during the experiment. This suggests that a concentration between 10 and 100 µM camptothecin would be required to impact the reproductive ability of *A. aegypti.*

TOP1 analogues are found in all eukaryotes and appear to be an essential enzyme during development in a wide variety of animals. During the process of DNA replication and transcription TOP1 is responsible for relaxing supercoiled DNA [44]. Knockouts of TOP1 are embryonically lethal in *Mus musculus* [45] and *Drosophila melanogaster* (Zhang, CX et al., 2000). TOP1 was demonstrated to be essential for larval and pupal growth, oogenesis and embryogenesis in *D. melanogaster* [46]. Larvae are developing and undergoing more growth, cell replication, and differentiation than in adults, which explains the greater susceptibility of larval stages that we observed.

Is it feasible to propose the use of a camptothecin derivative to target adult mosquitoes? We note the high concentrations of camptothecin (10 or 100 µM) that needed to be delivered in a blood meal to impact adult survival. Ivermectin is capable of killing *Anopheles* mosquitoes after they bite a human host who took the drug [47]. However the concentration of ivermectin required for lethality is in the low nanomolar range [48], and it is well tolerated and is used widely for mass drug administration (MDA) control of helminths in areas where mosquitoes and malaria are a problem [49]. We already discussed the potential antiviral use of camptothecin derivatives. Clearly compounds with improved potency against mosquitoes, as well as a much-improved safety profile, would need to be developed to be useful as anti-viral agents in humans with the additional benefit of controlling blood-feeding insect vectors.

## 4. Materials and Methods

### 4.1. Larval Motility Assay

*A. aegypti* egg papers were hatched in 500 mL pond salt solution (Blagon) supplemented with a quarter of a crushed 500 mg yeast tablet (Holland and Barrett), at 25 °C. After 18–24 h, larvae were collected with a 100 µm cell strainer, and diluted in pond salt solution to approximately 10 larvae per 100 µL.

Compounds were screened in 96-well plates. 100 μL of the larvae suspension was added to each well. There were approximately 10 larvae per well. The number of larvae per well did vary somewhat due to the stochastic nature of pipetting larvae from a stirred solution. However, the INVAPP system quantifies the overall movement within a well so was able to identify larvicidal compounds despite this variability.

For the primary screen using the Pandemic Response Box, the compound concentration in the assay was 10 µM, 1% *v*/*v* DMSO. Negative control (1% *v*/*v* DMSO) and positive control wells (10 µM deltamethrin, 1% *v*/*v* DMSO) were present on each assay plate. For the secondary screen, selected compounds were sourced from the original library material and screened at 10 µM, 1% *v*/*v* DMSO, with positive and negative controls as in the primary screen.

Movies were recorded and motility quantified using the INVAPP system [17,22]. Movies of 200 frames at 100 ms intervals were recorded immediately after the larval suspension was pipetted into the assay plates (nominally 0 h timepoint) and again after 2 h and 24 h.

The Pandemic Response Box was a gift from the Medicines for Malaria Venture. For the primary screen, each well was normalized for inhomogeneity in the number of mosquitoes dispensed per well by dividing the motility score at 2 h or 24 h by that of the same well at 0 h. The library was screened three times using independently prepared batches of mosquito larvae. Hit compounds were chosen where the median movement score at 2 h and/or 24 h was <40% of the same wells at 0 h.

The secondary screen was carried out on two occasions, each time with five independent assay plates (*n* = 10). The 24 h time point was analysed. For each assay plate, the median movement score of the negative and positive control replicate wells was calculated and used for subsequent analysis. The effect of compound treatment was determined using a one-way ANOVA test, and the identity of active compounds was then determined by Dunnett’s test, in comparison with that of the DMSO negative control.

Rubitecan, and the related compounds camptothecin and topotecan, were then re-tested using solid material at an assay concentration of 100 µM. Camptothecin (208925), rubitecan (9-nitrocamptothecin, R3655) and topotecan hydrochloride (T2705) were obtained from Merck Life Science.

Concentration response curves were fitted using the R package drc [50].

### 4.2. Adult Treatment Assays

10 mM camptothecin stock was made in DMSO. Blood containing 100 µM and 10 µM camptothecin, 1% and 0.1% DMSO (as solvent controls, respectively), and no additions (no DMSO control) were fed to 3 pools of 10 New Orleans adult (5–7 days old) females for each compound-concentration using a hemotek system. Adults were allowed 20 min to feed, and any unfed adults were removed. Adults were maintained in paper cups supplied ad libitum with 10% sugar solution on cotton wool.

At 4 days postblood-feeding, the surviving individuals (mortality recorded-0–96 h) were transferred to a 5 mL bijou tube with a 2.5 cm Whatman paper no.3 disk soaked in water pushed to the bottom to form a slight concave shape with a small pool of water for egg laying. Females were held in these tubes for 24 h to permit laying after which females were removed (mortality recorded: 96–120 h).

Lids were removed from tubes that were batch covered with netting to permit drying of the filter paper and eggs but preventing undesired egg laying by other mosquitoes. Seven days later, 2 mL of yeast suspension (1 yeast tablet dissolved/suspended in 500 mL water) was added to each tube to stimulate larval hatching and netting was replaced. Two days later, the number of larvae hatched, and number of eggs laid were counted.

Differences in lethality, the number of eggs and larvae, and the percentage of laid eggs that hatched were assessed with a Tukey HSD posthoc test in R.

## Data Availability

Data for the compound screen are provided in Table S1.

## Supplementary Materials

The following are available online. Video S1: Recording of *A. aegypti* larvae treated with camptothecin-derivatives at 100 µM, or the DMSO-only control, after 24 h. Images were recorded every 100 ms, Table S1: Effect of all MMV Pandemic Box compounds on *A. aegypti* larval motility after 2 and 24 h of treatment in the primary screen.

## Sample Availability

Camptothecin, rubitecan and topotecan are widely commercially available. Samples of other compounds found in the MMV Pandemic Response Box are not available from the authors but are distributed by Medicines for Malaria Venture (https://www.mmv.org/).
